# Supplementary material for: Analysis of 72,469 UK Biobank exomes links rare variants to male-pattern hair loss
Source: Nat Commun. 2023 Sep 22;14:5492. doi: 10.1038/s41467-023-41186-w (PMC10517150; doi:10.1038/s41467-023-41186-w)
Supplement: Supplementary file 1 — Supplementary Information [file 41467_2023_41186_MOESM1_ESM.pdf]

Supplementary Figures

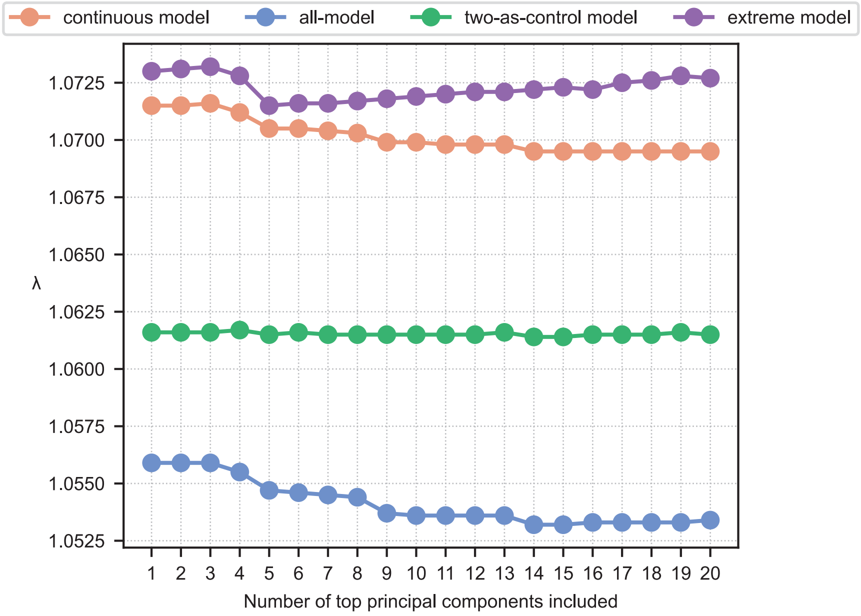

Supplementary Figure 1: Genomic inflation factor λ in GWAS with a varying number of included principal components. Genomic inflation factor λ according to the number of top principal components corrected for in a GWAS of imputed genotype data in the continuous model (orange), all-model (blue), the two-as-control model (green) and the extreme model (purple). The λ values generated were lowest when using 14-15 PCs in the continuous, all- and two-as-control models and 5 PCs in the extreme model, respectively.

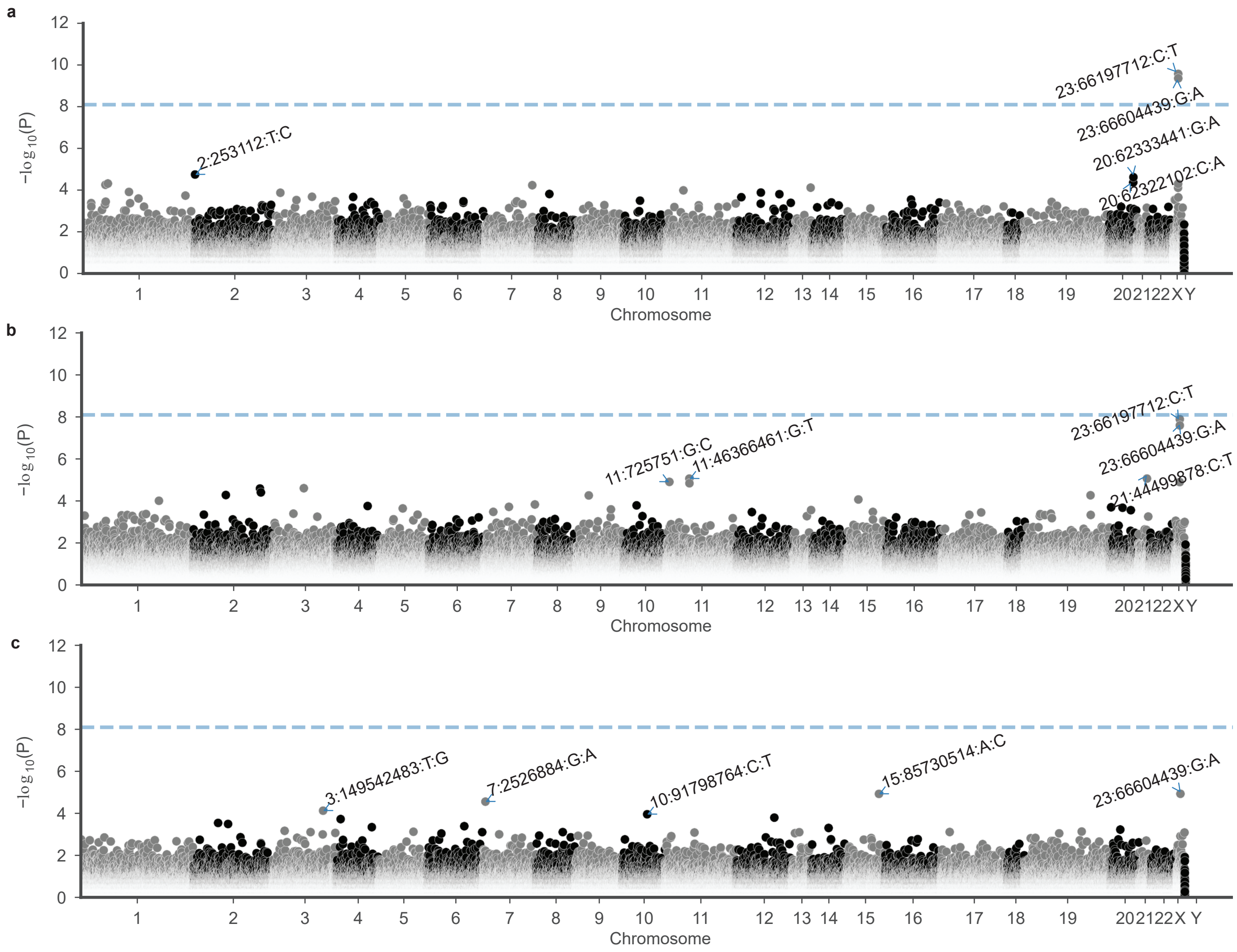

Supplementary Figure 2: Results of the single-variant analysis of additional phenotype models. Results are shown for **a** the all-model; **b** the two-as-control model; and **c** the extreme model. Only variants that were tested in the respective SKAT-O analysis are included. The dashed line denotes the selected genome-wide threshold for multiple testing in single-variant tests (8×10^{-9}). The y-axes depict −log10(P) obtained from logistic regression (two-sided, unadjusted). The top 5 variants per analysis were annotated.

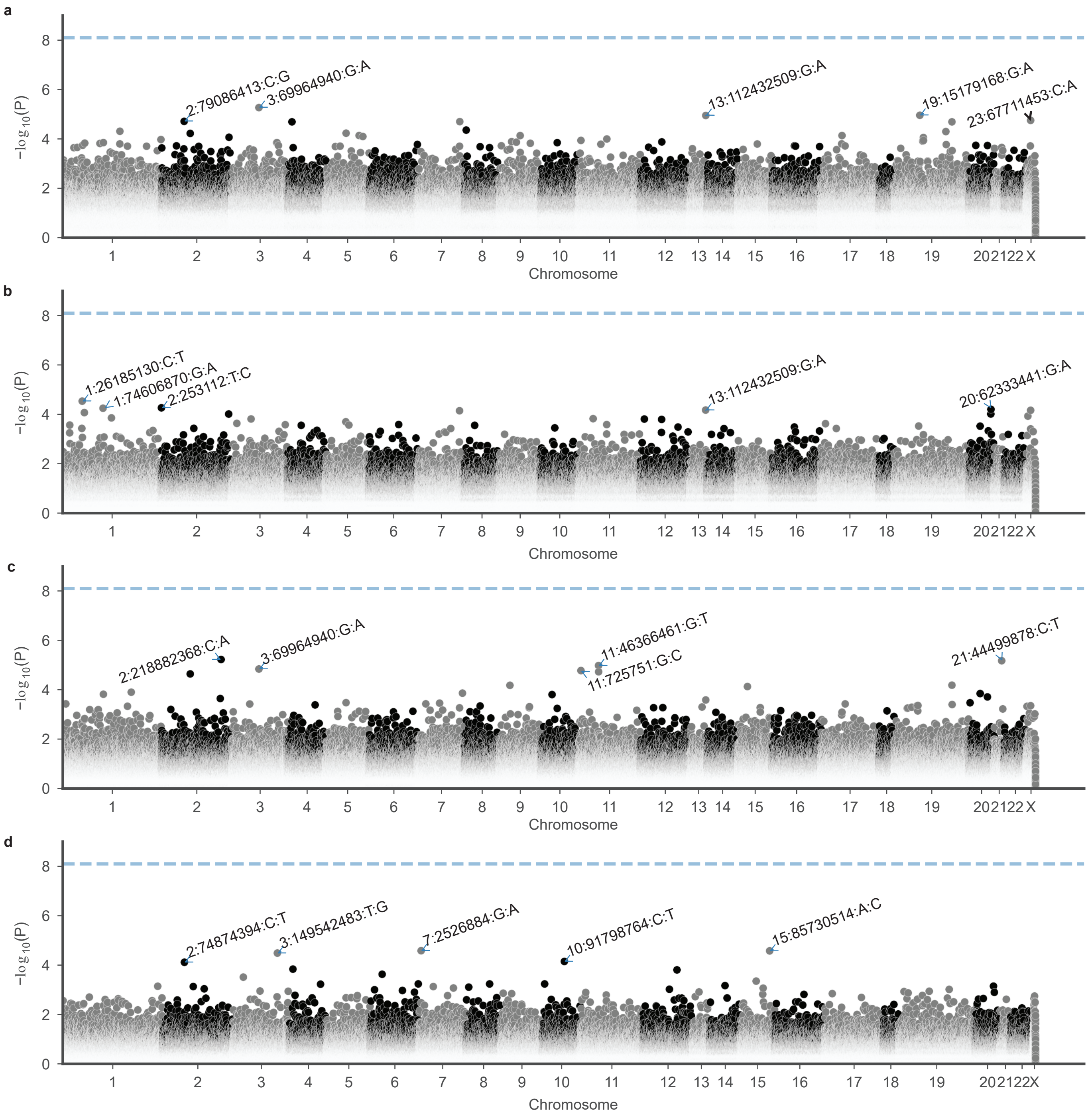

Supplementary Figure 3: Results of the single-variant analysis conditioned for 622 GWAS lead SNPs. Results are shown for **a** the continuous model; **b** the all-model; **c** the two-as-control model; and **d** the extreme model. Only variants that were tested in the respective SKAT-O analysis are included. The dashed line denotes the selected genome-wide threshold for multiple testing in single-variant tests (8×10^{-9}). The y-axes depict $-\log10$(P) obtained from linear regression (a) or logistic regression (b – d) (two-sided, unadjusted). The top 5 variants per analysis were annotated.

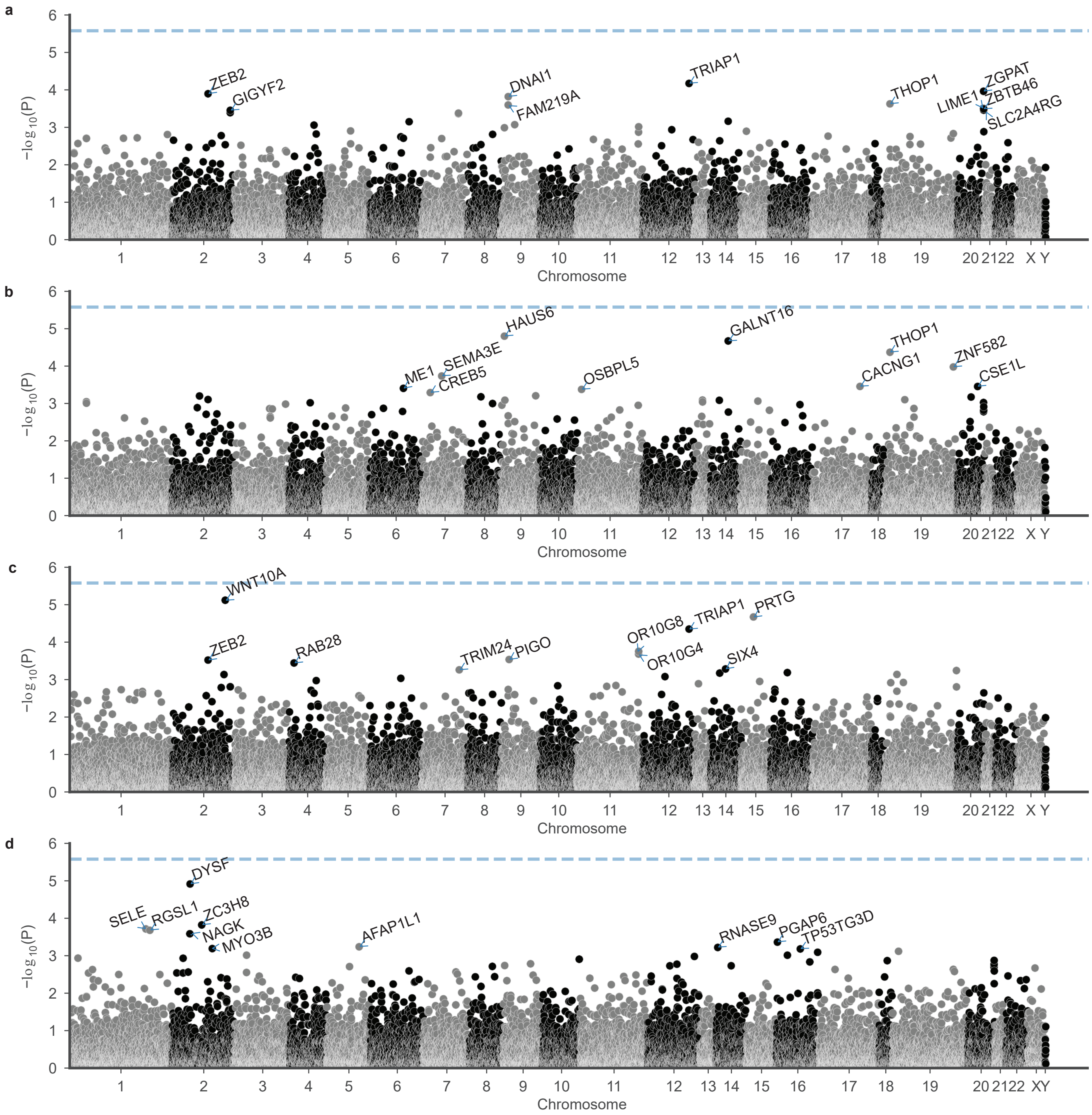

Supplementary Figure 4: Results of the SKAT-O gene-based analysis with a high impact variant threshold. Results are shown for **a** the continuous model; **b** the all-model; **c** the two-as-control model; and **d** the extreme model. The dashed line denotes the Bonferroni threshold for multiple testing in SKAT-O analyses (2.6×10^{-6}). The y-axes depict $-\log 10(P)$ obtained from linear regression (a) or logistic regression (b – d) (two-sided, unadjusted). The top 10 genes per analysis were annotated.

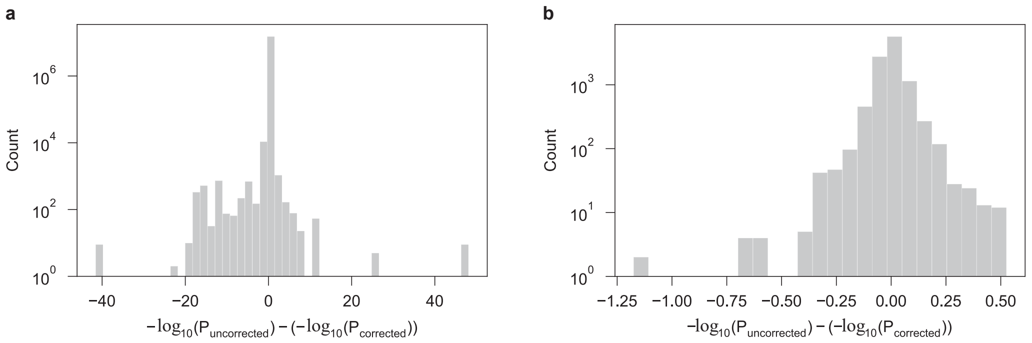

Supplementary Figure 5: Distribution of the decrease in $-\log_{10}$(P)-values in the conditional GWAS-GenRisk analysis. Distributions are shown across **a** all tested common variants and **b** GWAS lead SNPs. $P_{corrected}$ refers to the p-value generated when correcting for any single GenRisk gene score of a gene at the respective locus. Uncorrected and corrected p-values were generated by linear regression (two-sided, unadjusted).

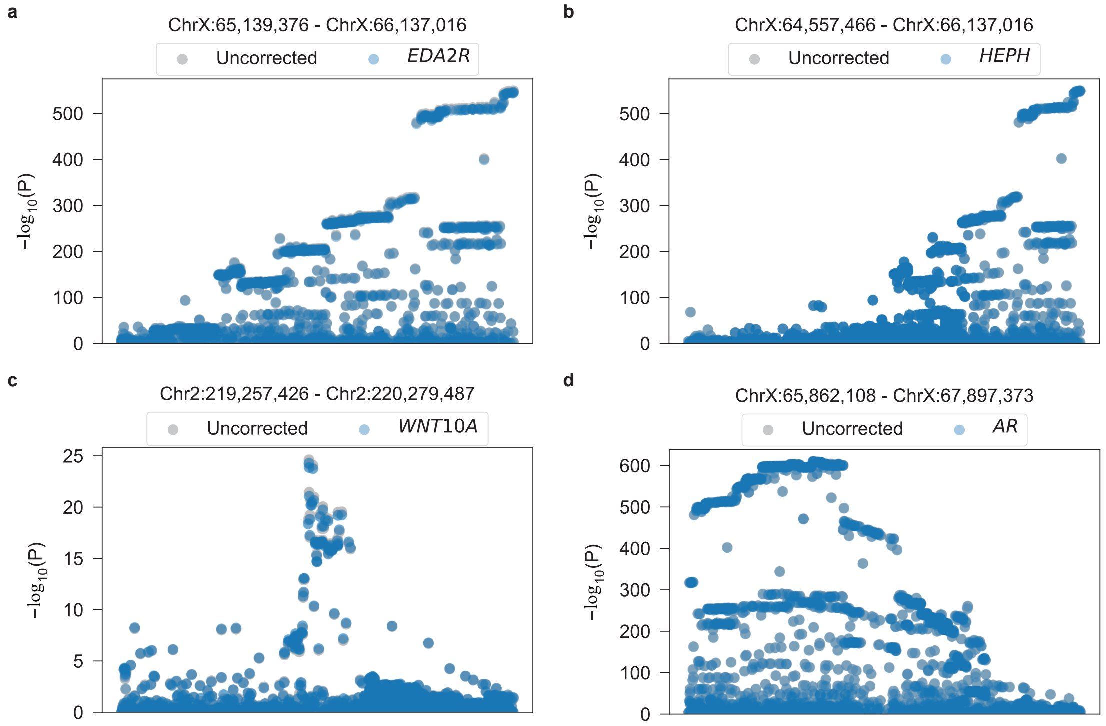

Supplementary Figure 6: Conditional GWAS-GenRisk results. Results are shown for the loci of **a** *EDA2R,* **b** *HEPH*, **c** *WNT10A*, and **d** *AR*. Association results without correction for GenRisk gene scores are shown in gray, association results after correction for GenRisk gene scores of a single gene are shown in blue, with the gene denoted in the respective legend. The y-axes depict $-\log 10$(P) obtained from linear regression (two-sided, unadjusted).

	Biological Process (Gene Ontology)			
GO-term	description	count in network	strength	false discovery rate
GO:0001942	Hair follicle development	6 of 74	1.28	0.0142

	Human Phenotype (Monarch)			
phenotype	description	count in network	strength	false discovery rate
HP:0000968	Ectodermal dysplasia	5 of 28	1.62	0.0026

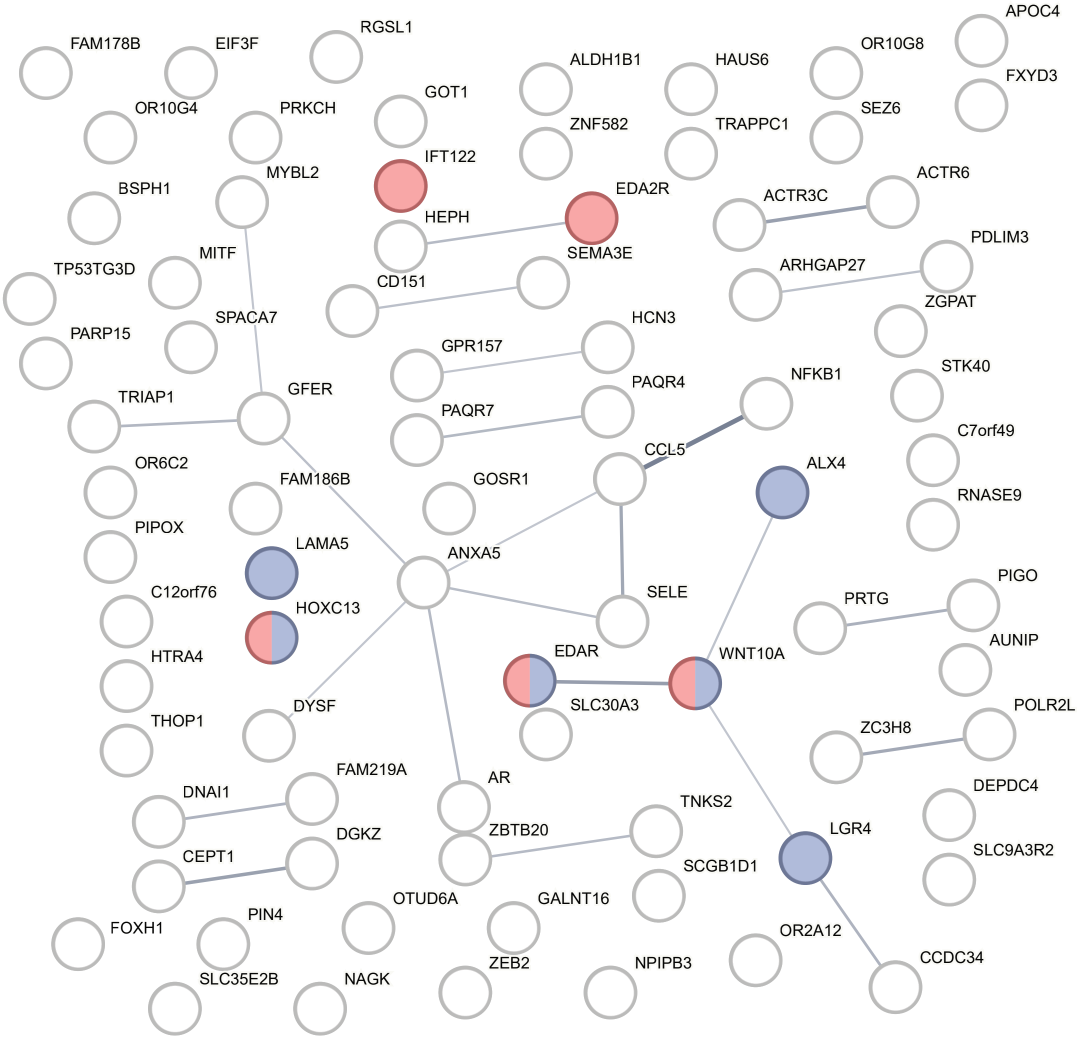

Supplementary Figure 7: Results of the STRING protein interaction network analysis of a less stringent set of MPHL-associated genes ($P < 3 \times 10^{-4}$ in the SKAT-O or GenRisk analyses). Functional enrichments performed by STRING based on one-sided hypergeometric tests are shown in the top panels, along with the corresponding false discovery rate (p-value corrected using the Benjamini-Hochberg method). Genes belonging to the enriched functional annotation terms are shown in blue (hair follicle development), orange (ectodermal dysplasia), or both. Line thickness indicates the strength of the data supporting the interaction.

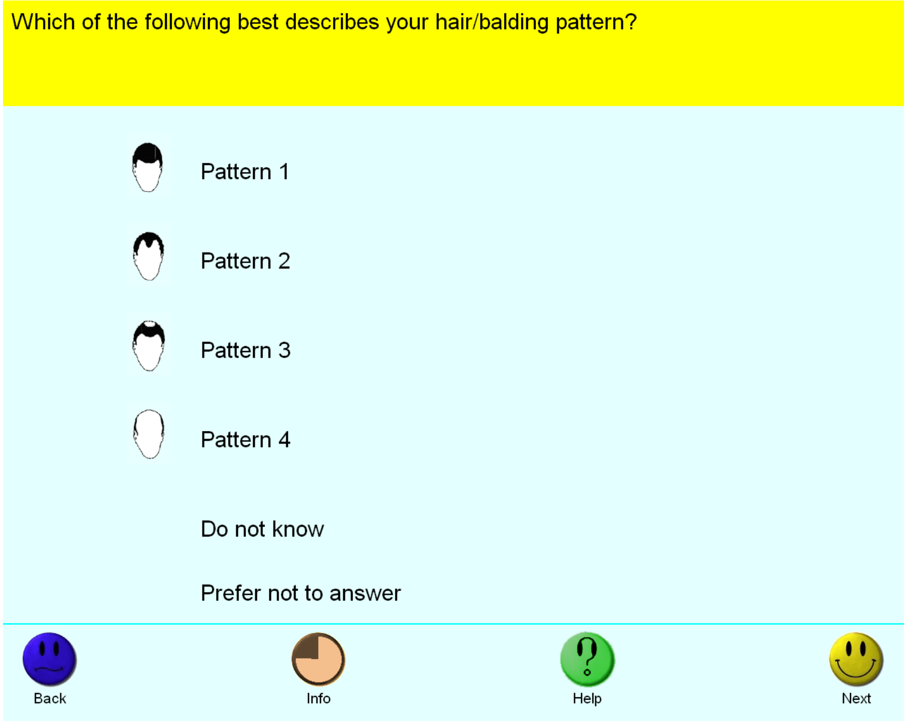

Supplementary Figure 8: Screenshot of the touchscreen questionnaire used to capture state of hair/balding pattern in the UK Biobank. Reproduced by kind permission of UK Biobank ©.

Supplementary Tables

Supplementary Table 1: List of known causative genes for monogenic trichoses, as used for enrichment testing.

Gene symbol	Ensembl ID	Gene name	Condition	References
ABCA5	ENSG00000154265	ATP Binding Cassette Subfamily A Member 5	Generalized hypertrichosis	[1]
ADAM17	ENSG00000151694	ADAM Metallopeptidase Domain 17	Structural hair defects	[2]
ALX4	ENSG00000052850	ALX Homeobox 4	Total alopecia in frontonasal dysplasia	[2]
ANTXR1	ENSG00000169604	ANTXR Cell Adhesion Molecule 1	Growth retardation, alopecia, pseudoanodontia (GAPO) syndrome	[2]
APCDD1	ENSG00000154856	APC Down-Regulated 1	Hypotrichosis 1	[1–3]
ATP7A	ENSG00000165240	ATPase Copper Transporting Alpha	Menkes disease	[2]
BCS1L	ENSG00000074582	BCS1 Homolog, Ubiquinol-Cytochrome C Reductase Complex Chaperone	Björnstad syndrome	[1,3]
C3orf52	ENSG00000114529	Chromosome 3 Open Reading Frame 52	Woolly hair/hypotrichosis	[1]
CDH3	ENSG00000062038	Cadherin 3	Ectodermal dysplasia, ectrodactyly, and macular dystrophy syndrome	[2–4]
CDSN	ENSG00000204539	Corneodesmosin	Hypotrichosis 2	[1–3]
CLDN1	ENSG00000163347	Claudin 1	Ichthyosis, leukocyte vacuoles, alopecia, and sclerosing cholangitis	[2]
DCAF17	ENSG00000115827	DDB1 And CUL4 Associated Factor 17	Woodhouse-Sakati syndrome	[2]
DLX3	ENSG00000064195	Distal-Less Homeobox 3	Trichodentoosseous syndrome	[2]

DSC3	ENSG00000134762	Desmocollin 3	Hypotrichosis and recurrent skin vesicles	[2,3]
DSG4	ENSG00000175065	Desmoglein 4	Hypotrichosis 6	[1–3]
DSP	ENSG00000096696	Desmoplakin	Carvajal syndrome	[1–3]
EDA	ENSG00000158813	Ectodysplasin A	Ectodermal dysplasia	[1–4]
EDAR	ENSG00000135960	Ectodysplasin A Receptor	Ectodermal dysplasia	[1–4]
EDARADD	ENSG00000186197	EDAR Associated Death Domain	Ectodermal dysplasia	[1–4]
EPS8L3	ENSG00000198758	EPS8 Like 3	Hypotrichosis 5	[1]
ERCC2	ENSG00000104884	ERCC Excision Repair 2, TFIIH Core Complex Helicase Subunit	Trichothiodystrophy	[2]
ERCC3	ENSG00000163161	ERCC Excision Repair 3, TFIIH Core Complex Helicase Subunit	Trichothiodystrophy	[2]
FGF13	ENSG00000129682	Fibroblast Growth Factor 13	Generalized hypertrichosis	[1,2]
FOXE1	ENSG00000178919	Forkhead Box E1	Hypothyroidism, with spiky hair and cleft palate	[2]
FOXN1	ENSG00000109101	Forkhead Box N1	T-cell immunodeficiency, alopecia, and nail dystrophy	[2]
GJB6	ENSG00000121742	Gap Junction Protein Beta 6	Clouston syndrome	[1,2]
GTF2H5	ENSG00000272047	General Transcription Factor IIH Subunit 5	Trichothiodystrophy	[2]
HOXC13	ENSG00000123364	Homeobox C13	Pure hair and nail ectodermal dysplasia	[1,2]

HR	ENSG00000168453	HR Lysine Demethylase And Nuclear Receptor Corepressor	Hypotrichosis 4, alopecia universalis	[2]
IKBKG	ENSG00000269335	Inhibitor Of Nuclear Factor Kappa B Kinase Regulatory Subunit Gamma	Incontinentia pigmenti, alopecia	[2,4]
JUP	ENSG00000173801	Junction Plakoglobin	Naxos disease	[1–3]
KRT25	ENSG00000204897	Keratin 25	Woolly hair/hypotrichosis	[1]
KRT71	ENSG00000139648	Keratin 71	Woolly hair	[1,2]
KRT74	ENSG00000170484	Keratin 74	Hypotrichosis 3, woolly hair	[1–3]
KRT75	ENSG00000170454	Keratin 75	Pseudofolliculitis barbae	[2,3]
KRT81	ENSG00000205426	Keratin 81	Monilethrix	[1–4]
KRT83	ENSG00000170523	Keratin 83	Monilethrix	[1–4]
KRT85	ENSG00000135443	Keratin 85	Pure hair and nail ectodermal dysplasia	[1–4]
KRT86	ENSG00000170442	Keratin 86	Monilethrix	[1–4]
LIPH	ENSG00000163898	Lipase H	Hypotrichosis 7	[1–3]
LPAR6	ENSG00000139679	Lysophosphatidic Acid Receptor 6	Hypotrichosis 8	[1–3]
LSS	ENSG00000160285	Lanosterol Synthase	Hypotrichosis 14	[1]
MBTPS2	ENSG00000012174	Membrane Bound Transcription Factor Peptidase, Site 2	Ichthyosis follicularis, atrichia, and photophobia syndrome	[2,3]
MPLKIP	ENSG00000168303	M-Phase Specific PLK1 Interacting Protein	Trichothiodystrophy	[2]

NECTIN1	ENSG00000110400	Nectin Cell Adhesion Molecule 1	Cleft lip/palate-ectodermal dysplasia syndrome	[1,4]
PADI3	ENSG00000142619	Peptidyl Arginine Deiminase 3	Uncombable hair syndrome	[5]
PKP1	ENSG00000081277	Plakophilin 1	Ectodermal dysplasia/skin fragility syndrome	[2,4]
PORCN	ENSG00000102312	Porcupine O-Acyltransferase	Goltz syndrome	[4]
RBM28	ENSG00000106344	RNA Binding Motif Protein 28	Alopecia, neurological defects, and endocrinopathy syndrome	[2,3]
RIN2	ENSG00000132669	Ras And Rab Interactor 2	Macrocephaly, alopecia, cutis laxa, and scoliosis	[2]
RPL21	ENSG00000122026	Ribosomal Protein L21	Hypotrichosis 12	[1,3]
SLC29A3	ENSG00000198246	Solute Carrier Family 29 Member 3	Histiocytosis-lymphadenopathy plus syndrome	[2,3]
SNRPE	ENSG00000182004	Small Nuclear Ribonucleoprotein Polypeptide E	Hypotrichosis 11	[1,2]
SOX18	ENSG00000203883	SRY-Box Transcription Factor 18	Hypotrichosis-lymphedema-telangiectasia syndrome	[2,3]
SOX9	ENSG00000125398	SRY-Box Transcription Factor 9	Hypertrichosis terminalis	[1,2]
SPINK5	ENSG00000133710	Serine Peptidase Inhibitor Kazal Type 5	Netherton syndrome	[2,3]
ST14	ENSG00000149418	ST14 Transmembrane Serine Protease Matriptase	Ichthyosis with hypotrichosis	[2,3]

TCHH	ENSG00000159450	Trichohyalin	Uncombable hair syndrome	[5]
TGM3	ENSG00000125780	Transglutaminase 3	Uncombable hair syndrome	[5]
TP63	ENSG00000073282	Tumor Protein P63	Ectodermal dysplasia	[1,2,4]
TRAF6	ENSG00000175104	TNF Receptor Associated Factor 6	Ectodermal dysplasia	[4]
TRPS1	ENSG00000104447	Transcriptional Repressor GATA Binding 1	Trichorhinophalangeal syndrome	[1,2]
VDR	ENSG00000111424	Vitamin D Receptor	Vitamin D-dependent rickets with alopecia	[2,3]
WNT10A	ENSG00000135925	Wnt Family Member 10A	Odonto-onycho-dermal dysplasia, Schopf-Schulz-Passarge syndrome	[1,2,4]

Supplementary References

1. Hayashi, R. & Shimomura, Y. Update of recent findings in genetic hair disorders. *J Dermatol* **49**, 55–67 (2022).

2. Duverger, O. & Morasso, M. I. To grow or not to grow: Hair morphogenesis and human genetic hair disorders. *Semin Cell Dev Biol* **25–26**, 22–33 (2014).

3. Betz, R. C., Cabral, R. M., Christiano, A. M. & Sprecher, E. Unveiling the roots of monogenic genodermatoses: Genotrichoses as a paradigm. *Journal of Investigative Dermatology* vol. 132 906–914 Preprint at https://doi.org/10.1038/jid.2011.408 (2012).

4. Wright, J. T. *et al.* Ectodermal dysplasias: Classification and organization by phenotype, genotype and molecular pathway. *Am J Med Genet A* **179**, 442–447 (2019).

5. Ü. Basmanav, F. B. *et al.* Mutations in Three Genes Encoding Proteins Involved in Hair Shaft Formation Cause Uncombable Hair Syndrome. *Am J Hum Genet* **99**, 1292–1304 (2016).
