# Supplementary material for: Analysis of 72,469 UK Biobank exomes links rare variants to male-pattern hair loss
Source: Nat Commun. 2023 Sep 22;14:5492. doi: 10.1038/s41467-023-41186-w (PMC10517150; doi:10.1038/s41467-023-41186-w)
Supplement: Supplementary file 3 — Description of Additional Supplementary Files [file 41467_2023_41186_MOESM3_ESM.pdf]

# Description of Additional Supplementary Files Document

**Supplementary Data 1:** Results of the single-variant and conditional single-variant association tests with at least nominal significance (P < 0.05). Conditional analysis was run on the same variants, but with MPHL-GWAS leadSNPs included as covariates on a per-chromosome basis.

**Supplementary Data 2:** Results of the SKAT-O association analyses. The labeled tabs each correspond to one phenotype model - variant consequence combination. Variant consequences are denoted as "nonsynonymous" (all nonsynonymous variants) or high (only variants classified as high impact according to Variant Effect Predictor). Per table, all tested genes and their respective p-value, FDR-adjusted p-value and variant / allele summary information are shown.

**Supplementary Data 3:** Results of the GenRisk association analysis. The labeled tabs each correspond to the results of one phenotype model. Per model, all tested genes and their respective test statistic, standard error, p-value and FDR-corrected p-value are shown. The GenRisk analysis was run on either all rare (MAF < 1%) variants, or on a filtered set of nonsynonymous coding variants, as used in the SKAT-O analysis (prefix 'coding_only').

**Supplementary Data 4:** Results of the conditional GWAS-Genrisk analysis filtered for associated variants whose p-value was impacted (|Δ-log<sub>10</sub>(P)| > 1) through correction for GenRisk gene scores. Variant positions are given in GRCh37.

**Supplementary Data 5:** ClinVar information on variants nominally significantly associated (P < 0.05) in any single-variant analysis, which were annotated as pathogenic or likely pathogenic in Clinvar, and their corresponding conditions. Also includes variants with conflicting interpretations of pathogenicity. Also shown is the prevalence of this variant in individuals per pattern group.

**Supplementary Data 6:** Results of the FUMA GENE2FUNC analysis of pathway gene sets. For each gene set, the overlap in relation to the pathway size, the test statistics before and after correction for multiple testing and the overlapping genes are shown. Pathway categories were filtered for canonical pathways, curated gene sets, computational gene sets, chemical and genetic perturbation, hallmark gene sets, Reactome, KEGG and Wikipathways.
