# Supplementary material for: Analysis of 72,469 UK Biobank exomes links rare variants to male-pattern hair loss
Source: Nat Commun. 2023 Sep 22;14:5492. doi: 10.1038/s41467-023-41186-w (PMC10517150; doi:10.1038/s41467-023-41186-w)
Supplement: Supplementary file 10 — Reporting summary [file 41467_2023_41186_MOESM10_ESM.pdf]

nature portfolio

Corresponding author(s): Dr. Stefanie Heilmann-Heimbach

Last updated by author(s): Jul 24, 2023

Reporting Summary

Nature Portfolio wishes to improve the reproducibility of the work that we publish. This form provides structure for consistency and transparency in reporting. For further information on Nature Portfolio policies, see our Editorial Policies and the Editorial Policy Checklist.

Statistics

For all statistical analyses, confirm that the following items are present in the figure legend, table legend, main text, or Methods section.

n/a	Confirmed	
☐	☒	The exact sample size (*n*) for each experimental group/condition, given as a discrete number and unit of measurement
☐	☒	A statement on whether measurements were taken from distinct samples or whether the same sample was measured repeatedly
☐	☒	The statistical test(s) used AND whether they are one- or two-sided<br>*Only common tests should be described solely by name; describe more complex techniques in the Methods section.*
☐	☒	A description of all covariates tested
☐	☒	A description of any assumptions or corrections, such as tests of normality and adjustment for multiple comparisons
☐	☒	A full description of the statistical parameters including central tendency (e.g. means) or other basic estimates (e.g. regression coefficient) AND variation (e.g. standard deviation) or associated estimates of uncertainty (e.g. confidence intervals)
☐	☒	For null hypothesis testing, the test statistic (e.g. *F*, *t*, *r*) with confidence intervals, effect sizes, degrees of freedom and *P* value noted<br>*Give P values as exact values whenever suitable.*
☒	☐	For Bayesian analysis, information on the choice of priors and Markov chain Monte Carlo settings
☒	☐	For hierarchical and complex designs, identification of the appropriate level for tests and full reporting of outcomes
☒	☐	Estimates of effect sizes (e.g. Cohen's *d*, Pearson's *r*), indicating how they were calculated

Our web collection on statistics for biologists contains articles on many of the points above.

Software and code

Policy information about availability of computer code

Data collection	No software was used for data collection.
Data analysis	Data processing and analyses were performed using publicly available software. PLINK v2.0 was used for data quality control and for variant-level association analyses. Variant annotation for the SKAT-O analysis was performed using the Ensembl Variant Effect Predictor (v104). Gene-level association analyses were performed using the SKAT v2.0.1 R package in R v4.0.5 and the GenRisk v0.2.5 Python module in Python v3.9.13. Downstream analyses included Fisher's exact tests performed using the scipy v1.8.1 Python module; polygenic risk score calculations using PRSice-2 v2.3.5; gene set enrichment using the FUMA v1.5.4 online resource; and protein-protein interaction network analysis using the STRING v11.5 online resource. AUC values for the PRS models of the full cohorts were computed using the pROC R package v1.18.

For manuscripts utilizing custom algorithms or software that are central to the research but not yet described in published literature, software must be made available to editors and reviewers. We strongly encourage code deposition in a community repository (e.g. GitHub). See the Nature Portfolio guidelines for submitting code & software for further information.

Data

Policy information about availability of data

All manuscripts must include a data availability statement. This statement should provide the following information, where applicable:

- Accession codes, unique identifiers, or web links for publicly available datasets
- A description of any restrictions on data availability
- For clinical datasets or third party data, please ensure that the statement adheres to our policy

This research has been conducted using data from UK Biobank under Application Numbers 24661 and 102444. The individual-level genetic and phenotypic data are available under restricted access; access can be obtained by application through the UK Biobank platform. The data generated that support the findings of this study are provided in the Supplementary Data. The CADD score data used in this study are available in the University of Washington CADD score database https://krishna.gs.washington.edu/download/CADD/v1.6/GRCh38/whole_genome_SNVs.tsv.gz. The gene feature annotation data used in this study are available in the Ensembl database under release number 104 https://ftp.ensembl.org/pub/release-104/gtf/homo_sapiens/Homo_sapiens.GRCh38.104.chr.gtf.gz and in the UCSC Genome Browser https://hgdownload.soe.ucsc.edu/goldenPath/hg38/database/refFlat.txt.gz. The ClinVar data used in this study are available from the ClinVar database https://ftp.ncbi.nlm.nih.gov/pub/clinvar/vcf_GRCh38/archive_2.0/2022/clinvar_20220430.vcf.gz.

Human research participants

Policy information about studies involving human research participants and Sex and Gender in Research.

Reporting on sex and gender	As male-pattern hair loss is a sex-limited trait that only affects individuals of male sex, the sample of this study comprised exclusively males of confirmed genetic sex (n=72,469). No information on participants' gender was available.
Population characteristics	The UK Biobank resource comprises volunteer participants from across the United Kingdom. For our sample set, we selected male individuals of white British ancestry (self-reported and based on a SNP-based principal components). The age of the participants in the final sample ranged from 39 to 82, with a mean age of 57.7 and a median age of 59.
Recruitment	The UK Biobank recruited volunteers living within 25 miles of one of 22 assessment centers throughout the United Kingdom. A number of biases have been well-documented, such as sex (biased towards female), age (skewed towards older ages) and health (biased towards healthier individuals).
Ethics oversight	The UK Biobank study has been approved by the North West Multi-centre Research Ethics Committee as a Research Tissue Bank and all participants provided written informed consent.

Note that full information on the approval of the study protocol must also be provided in the manuscript.

Field-specific reporting

Please select the one below that is the best fit for your research. If you are not sure, read the appropriate sections before making your selection.

☒ Life sciences ☐ Behavioural & social sciences ☐ Ecological, evolutionary & environmental sciences

For a reference copy of the document with all sections, see nature.com/documents/nr-reporting-summary-flat.pdf

Life sciences study design

All studies must disclose on these points even when the disclosure is negative.

Sample size	Our sample included a total of 72,469 male participants of the UK Biobank 200k exome sequencing data release. This represents the maximum number of unrelated male participants of white British descent with data of sufficient quality on genotype, exome sequence and baldness phenotype available in this data release.
Data exclusions	The UK Biobank 200k release contains exome and baldness data on 89,311 men. Of these, 557 were excluded during our quality control (mismatching reported and genetic sex, sex chromosome aneuploidy or implausible baldness self-report data). We further selected individuals of white British ethnicity (self-reported and based on SNP-based principal components), resulting in 74,620 individuals. Next, we excluded related individuals up to the third degree. In order to minimize the number of individuals excluded this way, we (i) iteratively excluded individuals with the highest number of related individuals and (ii) performed the relatedness exclusion separately for our extreme model, which comprised fewer individuals. A total of 72,469 men remained in the sample after these steps, of which 72,024 in the all- and two-as-control models and 17,053 in the extreme model.
Replication	To our knowledge, this is the first study testing for an association of rare coding variants with male-pattern hair loss in a large-scale exome sequencing data set. Currently, no other large-scale exome- or genome-sequencing data are available which would allow replication of this study. However, we identified a significant overlap of associated genomic loci with loci previously implicated through GWAS, which supports the validity of our findings.
Randomization	We controlled our association analyses for age and a specific number of top principal components (14 in the all- and two-as-control model

Randomization	and 5 in the extreme model). The correction for principal components aimed to account for population stratification within the sample, which we estimated by performing GWAS on imputed genotype data with age and a varying number (0-20) of top principal components included as covariates.
Blinding	Analysts were not blinded to participants' phenotype or case/control status. As the analyses were based on population-level observational data and the data were analyzed using automated computational methods of de-identified data, blinding was not relevant to the study.

Reporting for specific materials, systems and methods

We require information from authors about some types of materials, experimental systems and methods used in many studies. Here, indicate whether each material, system or method listed is relevant to your study. If you are not sure if a list item applies to your research, read the appropriate section before selecting a response.

Materials & experimental systems

n/a	Involved in the study
☒	☐ Antibodies
☒	☐ Eukaryotic cell lines
☒	☐ Palaeontology and archaeology
☒	☐ Animals and other organisms
☒	☐ Clinical data
☒	☐ Dual use research of concern

Methods

n/a	Involved in the study
☒	☐ ChIP-seq
☒	☐ Flow cytometry
☒	☐ MRI-based neuroimaging
